# Application of ALFA-Tagging in the Nematode Model Organisms *Caenorhabditis elegans* and *Pristionchus pacificus*

**DOI:** 10.3390/cells11233875

**Published:** 2022-12-01

**Authors:** Catia Igreja, Tobias Loschko, Alejandra Schäfer, Radhika Sharma, Shiela Pearl Quiobe, Elbin Aloshy, Hanh Witte, Ralf J. Sommer

**Affiliations:** Department for Integrative Evolutionary Biology, Max Planck Institute for Biology Tübingen, Max Planck Ring 9, 72076 Tübingen, Germany

**Keywords:** ALFA-tag, nematodes, *Caenorhabditis elegans*, *Pristionchus pacificus*, immunohistochemistry, immunoprecipitation

## Abstract

The detection, manipulation and purification of proteins is key in modern life sciences studies. To achieve this goal, a plethora of epitope tags have been employed in model organisms from bacteria to humans. Recently, the introduction of the rationally designed ALFA-tag resulted in a highly versatile tool with a very broad spectrum of potential applications. ALFA-tagged proteins can be detected by nanobodies, the single-domain antibodies of camelids, allowing for super-resolution microscopy and immunoprecipitation in biochemical applications. Here, we introduce ALFA-tagging into the two nematode model organisms *Caenorhabditis elegans* and *Pristionchus pacificus*. We show that the introduction of the DNA sequence, corresponding to the 13 amino acid sequence of the ALFA-tag, can easily be accommodated by CRISPR engineering. We provide examples of high-resolution protein expression in both nematodes. Finally, we use the GW182 ortholog *Ppa-ain-1* to show successful pulldowns in *P. pacificus*. Thus, the ALFA-tag represents a novel epitope tag for nematode research with a broad spectrum of applications.

## 1. Introduction

The introduction of various epitope tags has helped to transform research in biotechnology, as well as many other areas of the modern life sciences [1,2]. Short peptide epitope tags, such as Hemagglutinin (HA), FLAG^®^ and myc-tags, can be used to detect a protein of interest (POI) for which no antibody is available with minimal impact on protein function, and they also assist in (i) the purification of recombinant proteins in biotechnological applications, (ii) protein detection in cell and developmental biology and, finally, (iii) the immunoprecipitation of proteins—or macromolecular complexes— in biochemical studies with living cells or organisms. The recent implementation of ALFA-tag has resulted in a highly versatile tool for nanobody-based applications [3]. The ALFA-tag is a rationally designed 13 amino acid sequence that is hydrophilic and uncharged at physiological pH and devoid of residues that might react with fixatives. It forms stable α-helices that refold after chemical treatment and are compatible with protein function in living cells [3]. Importantly, an ALFA-tag can be added at any position in a POI. Given its sequence: Ser-Arg-Leu-Glu-Glu-Glu-Leu-Arg-Arg-Arg-Leu-Thr-Glu (SRLEEELRRRLTE), this tag is physically smaller than many others tags, and can be recognized by camelid single-domain nanobodies specifically targeting the ALFA peptide [3], further enhancing its performance in various applications.

Since its introduction in 2019, ALFA-tagging has been applied in various systems [3,4,5], but to the best of our knowledge not yet in nematodes. Nematodes represent one of the major animal phyla with an estimated species number of more than a million [6], with free-living, as well as animal and plant parasitic species [7]. *Caenorhabditis elegans* is an important nematode model system in many areas of the life sciences [8]. Several key characteristics of *C. elegans* fostered its use as a model organism. For example, *C. elegans* completes its development in 3–5 days (20 °C), can be cultured indefinitely on *Escherichia coli* bacteria on agar plates and propagates as self-fertilizing hermaphrodites, resulting in isogenic cultures [9]. Work over the last few decades has provided a detailed insight into the genetic and molecular mechanisms, among others, of *C. elegans* development, neurobiology and aging [8].

The enhanced understanding of the various biological processes in *C. elegans* has initiated comparative approaches in closely and more distantly related nematode species [10]. One species that has emerged as a powerful model system is *Pristionchus pacificus* [11,12]. Indeed, *P. pacificus* shares many functional tools with *C. elegans* and is another self-fertilizing species, with the two species thought to have diverged around 200 million years ago [13]. The development of CRISPR-associated technologies has also allowed precise manipulations of the nematode genomes [14,15], rapidly enhancing the application of various epitope tags. While the introduction of large fragments of DNA into the genome of *C. elegans* is easily achieved, size restriction of CRISPR-mediated genome editing is still a limitation in *P. pacificus* research [16]. Therefore, versatile, but physically short tags will be a major asset for research into this nematode.

Here, we apply ALFA-tagging in the two nematode models *C. elegans* and *P. pacificus*. Introduction of the DNA sequence corresponding to ALFA-tag can easily be accommodated by CRISPR engineering and we provide several examples of high-resolution protein expression in both species. Finally, we use the GW182 ortholog *Ppa-ain-1* to show successful pulldowns in *P. pacificus*, indicating that ALFA-tagging represents a new nematode tagging method with a broad spectrum of applications.

## 2. Materials & Methods

### 2.1. Maintenance of Worm Cultures

Stock cultures of *C. elegans* N2 and *P. pacificus* PS312 or RSC011 were reared at room temperature (20 °C) on nematode growth medium (NGM) in 6 cm Petri dishes, as originally described [9,11]. *Escherichia coli* OP50 were used as a food source. Bacteria were grown overnight at 37 °C in LB medium, and 200 μL of the overnight culture was pipetted onto NGM agar plates and left for several days at room temperature to grow bacterial lawns. *P. pacificus* PS312 worms were also grown in liquid cultures, as previously described [17]. Briefly, eggs-J1 animals from three fully grown plates obtained after bleach synchronization were grown in 10 mL of S medium [18] in autoclaved Erlenmeyer flasks with a volume of 50 mL, and containing a pellet of 100 mL overnight culture of *E. coli* OP50 grown in LB to an optical density (OD_600_) of 0.5. The liquid cultures were incubated at 22 °C with constant shaking (180 rpm) for 68 hrs and 72 hrs to obtain J4 and young adult worms, respectively.

### 2.2. Nomenclature of Tagged Genes and Proteins

We followed standard *C. elegans* and *P. pacificus* nomenclature rules. Tagged genes are referred to as i.e. *Cel-dlg-1(tu1782*[*dlg-1*::2xALFA]); tagged proteins are indicated as *Ppa-*EUD-1(tu1729[*Ppa*-EUD-1::2xALFA]).

### 2.3. CRISPR/Cas9 Mutagenesis

We followed a previously published protocol for *P. pacificus* with subsequently introduced modifications [15,19]. All target-specific CRISPR RNAs (crRNAs) were designed to target 20 bp upstream of the protospacer adjacent motifs (PAMs). Ten μL of the 100 μM stock of crRNA (CRISPR/Cas9 RNA; IDT) was combined with 10 μL of the 100 μM stock of tracrRNA (catalog# 1072534; IDT, USA), denatured at 95 °C for 5 min, and allowed to cool to room temperature and anneal. The hybridization product was combined with Cas9 protein (catalog# 1081058; IDT, USA) and incubated at room temperature for 5 min. The mix was diluted with Tris-EDTA buffer to a final concentration of 18.1 µM for the RNA hybrid and 2.5 µM for Cas9. For the induction of specific site-directed mutations via CRISPR/Cas9, an ssDNA oligo template was included in the mix at a concentration of 4 µM. The repair template (ssDNA donor) contained the desired modifications flanked by 35 nucleotide long homology arms at either side of the edited sequences. To avoid recutting of the edited allele, the PAM motif or the gRNA binding sequence included silent mutations. The ssDNA donor contained two copies of the sequence encoding the ALFA peptide codon optimized for *P. pacificus* and *C. elegans*. To minimize sequence identity, the two copies of the ALFA sequence in the ssDNA donor were designed with different codons for the same amino acid. The plasmid carrying the *Ppa-eft-3* promoter and the modified TurboRFP sequences [19] was used as co-injection marker. The sgRNAs, associated primers and repair templates for the specific targeted knock-ins utilized in this study can be found in Tables S1 and S2. The complete list of worm mutants generated in this study is present in Table 1.

Injections were performed on a Zeiss Axiovert microscope (Zeiss, Germany) coupled to an Eppendorf TransferMan micromanipulator and an Eppendorf FemtoJet injector (Eppendorf AG., Hamburg, Germany). The microinjection mixture was injected in the rachis of the gonad of an approximately one day old N2, PS312 and RSC011 (in the case of *Ppa ain-1(tu1753*[*ain-1*::2xALFA]) adult hermaphrodites (*n* = 40–50 *P. pacificus* worms; *n* = 30 *C. elegans* worms). Eggs laid by injected animals within a 12–16 h period post injection were recovered. After two days, the P0 plates containing the F1 (first generation) animals with fluorescent signals of co-injection marker were isolated, and 10–12 F1 progenies from each of the isolated P0 plates were singled out on individual plates. After the F1 animals had laid eggs, they were placed in 10 µL of single worm lysis buffer (10 mM Tris-HCl at pH 8.3, 50 mM KCl, 2.5 mM MgCl_2_, 0.45% NP-40, 0.45% Tween 20, 120 μg/ml Proteinase K), incubated in a thermocycler at 65 °C for 1 h, followed by heat deactivation of the proteinase at 95 °C for 10 min. The resulting lysate was used as a template in subsequent PCR steps. The genotype of the F1 animals were subsequently analyzed via Sanger sequencing and any mutations were identified before re-isolation in homozygosis. In *P. pacificus*, the CRISPR/Cas9 mediated knock-in efficiency varied between 1–3%.

### 2.4. Mouth-Form Phenotyping in P. pacificus

*P. pacificus* mouth-form phenotyping was performed as previously described [20]. Briefly, 30 adult hermaphrodite worms were mounted onto slides with 5% Noble Agar pads with 0.3% NaN_3_ added as an anesthetic and examined using DIC microscopy. Individual worms were distinguished by the presence (Eurystomatous, Eu) or absence (Stenostomatous, St) of a large right sub ventral tooth and a curved dorsal tooth. The number of biological replicates (*n*) was ≥3 for all conditions, with each replicate comprising at least 30 animals. Error bars represent the standard deviation.

### 2.5. ALFA-Tag Immunostaining

Healthy (not starved) and fully grown worms were collected from 6 cm Petri dishes (*n* = 10) with M9 medium or from liquid cultures and filtered through a 5 µm Nylon net filter (Milipore Merck, Darmstadt, Germany), as previously described [17]. All *P*. *pacificus* developmental stages were retained by the 5 µm filter, while bacteria passed through. Worms were fixed with 4% para-formaldehyde, 1xPBS (500 µL) in 1.5 mL Eppendorf tubes overnight on a tube rotator. After fixation, the samples were washed three times with 0.5% Triton X-100, 1x PBS (washing solution). To chemically reduce the disulfide bonds between collagen fibers in the cuticle and to enhance the diffusion of the chemical reagents into the specimen, animals were incubated overnight in 500 µL of buffer containing 5% β-mercaptoethanol, 1% Triton X-100 and 0.1 M Tris-HCl pH 7.4, with gentle shaking (300 rpm). Following two washes with 1% Triton X-100, 0.1 M Tris-HCl pH 7.4 buffer and one wash with collagenase buffer (1 mM CaCl_2_, 1% Triton X-100, 0.1 M Tris pH 7.4), worms were digested in 200 µL of collagenase buffer with 200 units of collagenase type IV (Gibco, Thermo Fisher Scientific, Waltham, MA, USA) at 37 °C for 2–3 hrs or until the thinning of the cuticle caused the worms to break at the vulva region. Afterwards, the samples were washed three times with the washing solution. Digested animals were then incubated for 1 h at RT in 100 µL of blocking solution (1% BSA, 0.5% Triton X-100, 1x PBS). After two washes in washing solution, animals were stained with the primary antibody (FluoTag-X2 anti-ALFA-AbberiorStar580, NanoTag Biotechnologies, Göttigen, Germany, 1:100) in 50 µL of blocking solution, overnight at 4 °C in a tube rotator. Worms were washed thrice with the washing solution, resuspended in VectaShield mounting medium (Vector Laboratories, USA) containing 1 μg/mL 4′,6-diamidino-2-phenylindole (DAPI; Molecular Probes, Thermo Fisher Scientific) and gently mounted on freshly prepared 5% Noble agar pads. Images were acquired on a Leica TCS SP8 microscope and assembled on Fiji (Image J, NIH, Bethesda, MD, USA).

### 2.6. ALFA-Tag Immunoprecipitation

*P. pacificus* PS312 and ALFA-tagged AIN-1 (*Ppa-ain-1(tu1753*[*ain-1*::2xALFA]) worms were collected from 10 cm Petri dishes (*n* = 100) with M9 medium and filtered through a 5 µm Nylon net filter (Milipore Merck, Darmstadt, Germany), dropped into liquid nitrogen to produce frozen “worm popcorns/pellets”, and stored at –80 °C. The worm pellets were ground into a fine powder using a mortar and pestle pre-cooled with liquid nitrogen and the powder was then transferred to a Falcon tube with 10 mL of cold egg buffer (118 mM NaCl, 48 mM KCl, 2 mM CaCl_2_, 2 mM MgCl_2_, 25 mM HEPES, pH 7.3) [21]. This worm suspension was centrifuged at 2000 rpm, 4 °C for 2 min, and the resulting worm pellet was resuspended in 8 mL of lysis buffer [50 mM Tris-HCl pH7.5, 150 mM KCl, 5 mM MgCl_2_, 2 mM EDTA, 0.1% Triton X-100, 2 mM DTT, 10% glycerol, complete protease inhibitor (Roche, Switzerland), PMSF)] after discarding the supernatant. The worm solution was homogenized with a Wheaton Dounce Dura-Grind tissue grinder (DWK Life Sciences, Mainz, Germany) (10 strokes) to break the cuticle. The lysate was cleared from full worms by centrifugation (100 g, 4 °C, 6 min); the supernatant was transferred to a fresh 15 mL Falcon tube and the remaining pellet was resuspended in 8 mL of lysis buffer and further ground on the Wheaton Dounce with 25 strokes. Following a short centrifugation to remove non-homogenized worms (100 g, 4 °C, 6 min), the supernatants (worm lysates) were combined into one tube and cleared with a high-speed centrifugation (19,000 g, 4 °C, 20 min). The clarified extract was transferred to a fresh tube and input samples were collected for western blotting. To reduce non-specific protein binding to the beads, we precleared the lysate with Protein G sepharose resin (75 μL of 50% slurry) for an hour at 4 °C in a tube rotator. After centrifugation at 1000 g for 2 min, the supernatant was transferred to a fresh tube containing 75 μL of previously equilibrated ALFA selector^PE^ resin (NanoTag Technologies, Göttigen, Germany) and incubated for 1–2 hrs at 4 °C in a tube rotator. The beads were sedimented by centrifugation (1 min, 1000 g, 4 °C) and washed thrice with the lysis buffer. To elute the ALFA-tagged proteins, the beads were resuspended in 200 μL of lysis buffer, transferred to a Mini Spin column (NanoTag Biotechnologies, Göttigen, Germany) and centrifuged for 1 min at 1000 g. The flow-through was discarded and the beads were washed twice with 400 μL of lysis buffer without DTT, glycerol and protease inhibitor and once with TBS 1x. The Mini Spin column was capped with a bottom plug and transferred to a fresh Eppendorf tube. Elution of the ALFA protein was performed with five column bed volumes of elution buffer (400 μM of the ALFA peptide in 200 μL of lysis buffer) for 20 min at room temperature with subtle shaking. The Mini Spin column bottom plug was removed to collect the eluate by centrifugation (1 min, 1000 g, 4 °C). To achieve the highest yields, the elution step was repeated and the eluates were combined. An aliquot of the eluate was collected for analysis by Western blot. To maximize the elution efficiency, an additional elution step with a denaturing sample buffer (NuPAGE LDS sample buffer, Life Technologies, Carlsbad, CA, USA) can also be performed on the ALFA resin.

### 2.7. Western Blot

Western blot was performed using standard methods. Briefly, worms were collected from 6 cm Petri dishes (*n* = 10) with M9 medium and filtered through a 5 µm Nylon net filter (Milipore Merck, Darmstadt, Germany), as previously described [17]. The worm pellet was flash-frozen in liquid nitrogen and stored at –80 °C. To obtain worm extracts, frozen worm pellets were ground to a powder on a mortar and pestle pre-cooled with liquid nitrogen before the addition of 2X Laemmli Sample Buffer (100 mM Tris-HCl pH = 6.8, 4% SDS, 20% glycerol, 0.2 M DTT; 3x the volume of the worm pellet). Samples were stored at −20 °C prior to the analysis. Before SDS-PAGE, the samples were boiled for 5 min at 95 °C and vortexed to shear genomic DNA. Proteins were transferred from the SDS-PAGE gel onto a nitrocellulose membrane (Santa Cruz Biotechnology, Dallas, TX, USA) by tank transfer. Primary antibodies were incubated overnight at 4 °C, secondary antibodies for an hour at room temperature in blocking solution (5% milk, 0.3% Tween 20, PBS 1x). All Western blots were developed with Clarity Western ECL substrate solutions (Bio-Rad, USA). The antibodies used in this study are as follows: sdAB anti-ALFA-HRP (N1505-HRP, NanoTag Biotechnologies, Göttigen, Germany, 1:1000), mouse monoclonal anti-Tubulin (Sigma Aldrich, St. Louis, MI, USA, T6199, 1:500), rabbit anti-GRP78 (Thermo Fisher Scientific, USA, PA5-22967, 1:1000), sheep polyclonal anti-mouse IgG-HRP (GE Healthcare, Chicago, IL, USA, RPN4201, 1:5000) and anti-rabbit IgG-HRP (GE Healthcare, USA, NA934V, 1:5000).

### 2.8. Mass Spectrometry Measurements

For analysis of the *Ppa-*AIN-1::2xALFA interactome, following the in-gel protein digestion with trypsin of the two replicas of PS312 and AIN-1::2xALFA elutes, the peptides were separated and analyzed on a Proxeon Easy-nLC 1200 UHPLC (Thermo Fisher Scientific, USA) coupled to a Q Exactive HF mass spectrometer (Thermo Fisher Scientific, USA). Eluted peptides were ionized on an electrospray ionization source. The MS data acquisition was conducted in the positive ion mode and data-dependent acquisition mode. The 12 most intense multiple-charged ions were selected for higher-energy collision dissociation (HCD) fragmentation. Raw data files were processed with the MaxQuant software suite v.1.5.2.28 [22,23]. Using the Andromeda search engine integrated in the software, the spectra were searched against the Uniprot *P. pacificus* complete proteome database and a database comprising the ALFA tag sequence and commonly observed contaminants. The processing of the raw data was performed with a setting of 1% for the FDR (False Discovery Rate).

## 3. Results and Discussion

### 3.1. CRISPR-Based ALFA Tagging in C. Elegans and P. Pacificus

We selected a total of five genes in *C. elegans* and *P. pacificus* to generate ALFA-tagged fusion proteins in both species (Table 1). For all genes, we added two copies of the ALFA-tag sequence, resulting in an addition of 78 nucleotides to the coding region of the gene of interest that translated into an extra 26 amino acids in the protein. Specifically, in *C. elegans* we chose *dlg-1* (*discs large-1*), a membrane-associated guanylate kinase-like protein that is localized to the adherens junctions of all epithelia [24,25], and *unc-10* which is expressed at synapse-dense regions, such as the nerve ring and the dorsal and ventral nerve cords [26]. *Cel-*UNC-10 is the nematode homolog of the Rab3-interacting molecule (RIM)-binding protein and is involved in the presynaptic localization of the voltage-gated calcium channel (VGCC) UNC-2 [27]. The expression pattern of both of these proteins has been characterized in multiple publications via the use of monoclonal antibodies or GFP-transgenic animals [24,25,26,28]. We tagged both genes at the C-terminus, immediately before the stop codon (Table 1). In *P. pacificus*, we targeted two mouth-form regulatory genes, *Ppa-eud-1* and *Ppa-seud-1/sult-1*, encoding for proteins involved in sulfation processes [29,30,31,32], and *Ppa-ain-1,* the nematode homolog of the Argonaute interacting protein GW182, which is known to be broadly expressed [33,34]. Spatial expression profiling of the *Ppa-*EUD-1 sulfatase and the sulfotransferase *Ppa-*SEUD-1/SULT-1 has been determined using fluorescent transcriptional reporters or fluorescent in situ hybridization (FISH) [29,30,31,35,36,37]. The three *P. pacificus* proteins were also C-terminally tagged (Table 1).

**Figure 1:**
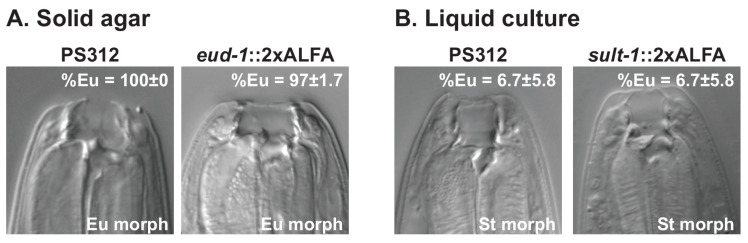
EUD-1 and SULT-1 ALFA tagging in *P. pacificus* (**A**,**B**) Images depicting the eurystomatous (Eu) (**A**) or stenostomatous (St) (**B**) morphs of PS312, *eud-1*::2xALFA and *sult-1*::2xALFA animals grown on agar plates (**A**) and liquid cultures (**B**). Eu worms have a wide-mouth with two teeth. The bacterial feeding St worms show a narrow mouth with a single tooth. The numbers in the images represent the mean value ± the standard deviation of mouth–form ratio in the different environmental conditions and worm strains presented as percent Eu (% Eu).

CRISPR-engineering worked efficiently for all five targeted genes with all the resulting lines being viable. All the data shown in this manuscript was obtained with CRISPR-engineered worm strains that resembled the parental wild-type strains, i.e., the worms containing ALFA-tagged proteins were without any visible abnormal phenotype. As changes in the function of the sulfation-related enzymes affect mouth-form ratios in *P. pacificus* [29,31], we determined the Eu/St mouth-form ratios of the *Ppa-eud-1(tu1729*[*eud-1*::2xALFA]) and *Ppa-sult-1(tu1760*[*sult-1*::2xALFA]) strains, when grown on solid agar plates and liquid cultures, respectively. Similar to the laboratory California strain (PS312) of *P. pacificus*, *Ppa-eud-1(tu1729*[*eud-1*::2xALFA]) adult worms were mainly Eu (97.7 ± 1.7% Eu: Figure 1A) when grown on solid agar. In contrast, PS312 and the *Ppa-sult-1(tu1760*[*sult-1*::2xALFA]) worms developed largely into St morphs in liquid culture (only 6.7 ± 5.8 % Eu; Figure 1B), as previously observed [17]. Thus, CRISPR-based ALFA tagging in *C. elegans* and *P. pacificus* did not interfere with protein function.

### 3.2. High-Resolution Immunofluorescence of ALFA-Tagged Proteins in C. Elegans

**Figure 2:**
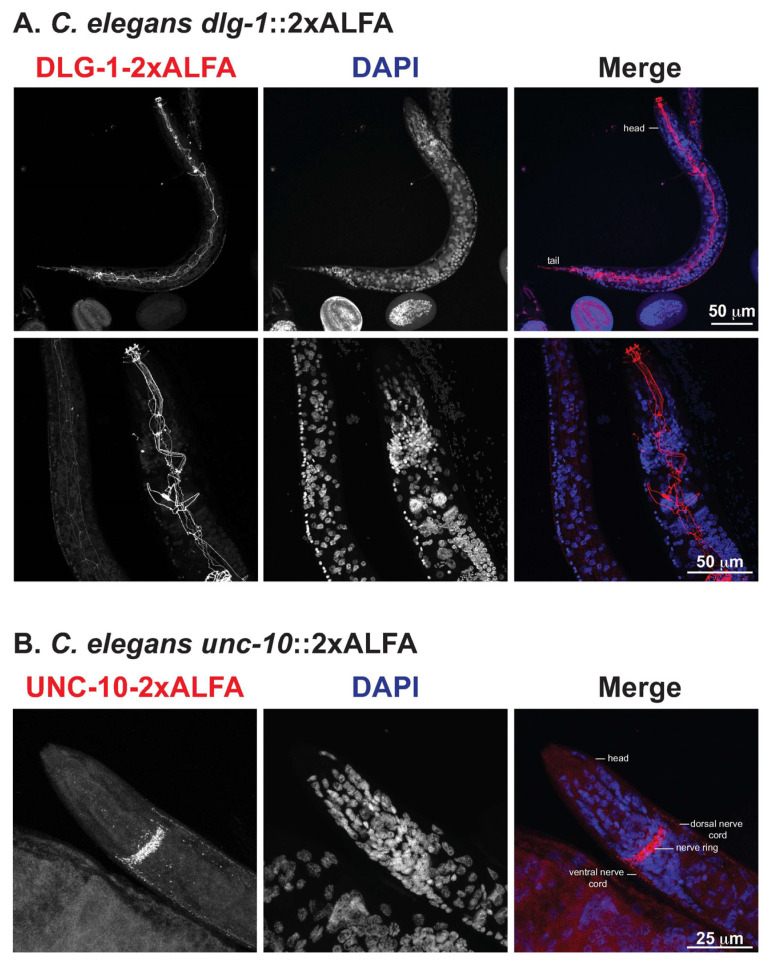
Spatial expression patterns of *Ce dlg-1*::2xALFA and *Ce unc-10*::2xALFA in adult worms. (**A**,**B**) Representative confocal fluorescent micrographs of the body and head of fixed *C. elegans dlg-1(tu1782*[*dlg-1*::2xALFA]) (**A**) and *unc-10(tu1781*[*unc-10*::2xALFA]) (**B**) animals. The worms were stained with anti-ALFA fluorescent nanobody. The merged images show the anti-ALFA signal in red and the nuclei in blue (DAPI staining). Images depict L4/adult worms.

We first analyzed the expression of *Cel-*DLG-1 using the *dlg-1*::2xALFA line. In adult animals, we observed strong staining of *Cel-*DLG-1::2xALFA in epithelial cells of the epidermis, pharynx and intestine, forming a continuous belt around the cells from the head to the tail (Figure 2A, upper panels). In the head region of adult hermaphrodites, *Cel-*DLG-1::2xALFA is seen in the pharynx with a high resolution of the protein localization (Figure 2A, bottom panels). The observed expression is similar to previous reports [25] and is present throughout worm development (data not shown), which indicates the high structural-resolution obtained by visualization with nanobodies. In contrast, *Cel-*UNC-10::2xALFA staining was restricted to the synapse-rich areas of the nervous system, including the nerve ring and the dorsal and ventral nerve cords (Figure 2B), as previously reported [26]. These data demonstrate the specificity of the anti-ALFA nanobody. Taken together, ALFA-tagging is a simple and reliable method for high-resolution in situ protein detection, and it can be combined with the analysis of transcript expression in order to provide a comprehensive comparison of gene expression at all levels.

### 3.3. Visualization of ALFA-Tagged Proteins in P. pacificus Reveals Sub-Localization of Mouth-Form Regulators

Next, we wanted to generate ALFA-tagged proteins in *P. pacificus* to study if this type of epitope tag works across nematode species borders. Note that the implementation of several methodologies that work routinely in *C. elegans* had proven difficult in other nematodes. In *P. pacificus*, for example, the establishment of DNA-mediated transformation required major modifications of the original protocol and took more than a decade [38]. In contrast, CRISPR-mediated gene knock-out was easily transferred from *C. elegans* [39], although this was later improved by several laboratories [15,19].

**Figure 3:**
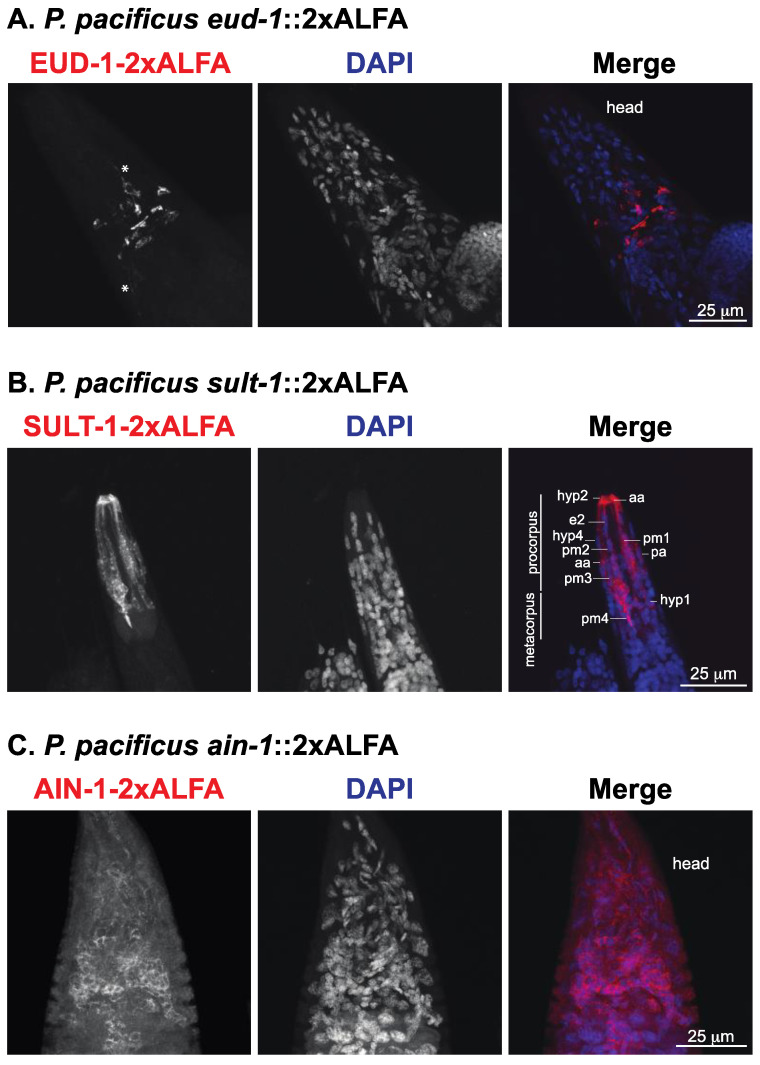
ALFA-tagged proteins show distinct expression patterns in *P. pacificus* (**A–C**) Representative confocal fluorescent micrographs of the head of fixed *P. pacificus eud-1(tu1729*[*eud-1*::2xALFA]) (**A**), *sult-1(tu1760*[*sult-1*::2xALFA]) (**B**) and *ain-1(tu1753*[*ain-1*::2xALFA]) (**C**) animals. The worms were stained with anti-ALFA fluorescent nanobody. The merged images show the anti-ALFA signal in red and the nuclei in blue (DAPI staining). To maximize the expression of the sulfotransferase, *sult-1(tu1760*[*sult-1*::2xALFA]) animals were grown under liquid culture conditions. Images depict J4/adult worms. In A, asterisks indicate neuronal projections. In B, cell types were identified based on the reconstruction of the head hypodermis in *P. pacificus* [40]. aa: anterior arcade cell, e: epidermal cell, hyp: hypodermal syncytia, pm: pharyngeal muscle cell.

We used the two sulfation-associated proteins *Ppa-*EUD-1 and *Ppa-*SEUD-1/SULT-1, representing a sulfatase and sulfotransferase, respectively. In agreement with the spatial information revealed by fluorescent transcriptional reporters [29,31,35] and FISH^36^, *Ppa-*EUD-1::2xALFA protein was detected in different somatic and pharyngeal neurons in the head of the animals (Figure 3A). The higher resolution of the anti-ALFA nanobody also revealed the expression of *Ppa-*EUD-1::2xALFA in the long axonal processes leading towards the mouth opening or the excretory gland cell (Figure 3A, asterisk). At the anterior of the worm, SEUD-1/SULT-1::2xALFA was expressed in cells that make up the face (anterior body wall), the stoma (mouth), the anterior pharynx (Figure 3B) in the feeding apparatus of *P. pacificus*. The *Ppa-*SEUD-1/SULT-1::2xALFA protein was preferentially detected in experimental conditions that promote the St morph (Figure 1B). *Ppa-*SEUD-1/SULT-1 was present in the cytoplasm of cells of the anterior epidermal (hyp) syncytia, the arcade cells at the interface of the mouth and the pharynx, and the pharyngeal muscle cells that secrete the extracellular teeth of the animal (Figure 3B), as previously observed [30,31,40].

Finally, we targeted the nematode ortholog of the human GW182 gene *Ppa-ain-1*, a broadly expressed protein that localizes as discrete cytoplasmic ribonucleoprotein particles known as processing (P)-bodies [33]. Indeed, we observed that *Ppa*-AIN-1::2xALFA was a cytoplasmic protein expressed in the majority of the cells in adult worms (Figure 3C). Based on the fact that the analyzed ALFA-tagged proteins show distinct spatial expression patterns, which is in agreement with prior studies, we conclude that ALFA-tagging allows high-resolution and specific in situ visualization of proteins in *P. pacificus* and *C. elegans*.

### 3.4. Detection of ALFA-Tagged Proteins by Western Blot

**Figure 4:**
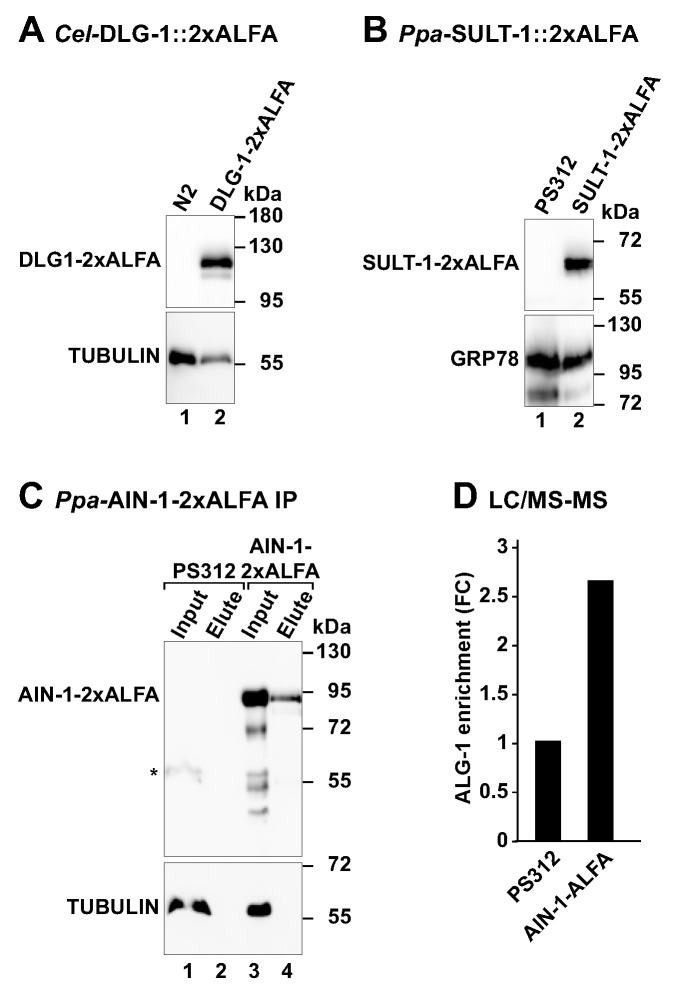
Immunoblotting and pulldown of ALFA-tagged proteins (**A**,**B**) Western blot showing the expression levels of the ALFA-tagged proteins in the respective worm strains. *C. elegans* N2 and *P. pacificus* PS312 worms were used as mock controls for the absence of ALFA-tagged proteins. The blots were developed with anti-ALFA nanobody directly coupled with horse radish peroxidase (HRP) or with a mouse anti-TUBULIN primary antibody followed by anti-mouse IgG-HRP antibody. Molecular weight markers (kDa) are shown next to the blot. (**C**) Affinity purification of *Ppa*-AIN-1::2xALFA using ALFA resins. *Ppa-ain-1(tu1753*[*ain-1*::2xALFA]) worm lysates were incubated with ALFA selector^PE^ resin. After washing, the resin was incubated with 400 μM of ALFA peptide to elute the *Ppa*-AIN-1::2xALFA bound to the resin. Input (30% of worm lysate) and elute (2.5% of eluted *Ppa*-AIN-1::2xALFA protein) fractions were analyzed by SDS-PAGE and Western blot. The membrane was first incubated with anti-TUBULIN primary antibody and the corresponding secondary antibody coupled with HRP. After acquisition of the TUBULIN signal, ALFA-tagged protein was detected using the anti-ALFA nanobody. The control reaction (no ALFA-tag on AIN-1) was set with *P. pacificus* PS312 worm lysate. The asterisk indicates the signal remaining from the TUBULIN protein. (**D**) Bar plot showing the difference (fold change) in the number of *Ppa*-ALG-1 peptides present in duplicated samples of *Ppa-*AIN-1::2xALFA and wild type (PS312) worm strains quantified using LC/MS-MS.

We then tested the nematode ALFA tagged proteins using Western blot. Animals from *Cel-dlg-1(tu1782*[*dlg-1*::2xALFA]), *Ppa*-*sult-1(tu1760*[*sult-1*::2xALFA]) and *Ppa*-*ain-1(tu1753*[*ain-1*::2xALFA]) worms were collected, lysed and used in SDS-PAGE. Wild type N2 and PS312 worms were included as control lysates lacking ALFA-tagged proteins. All worm lysates were probed with the nanobody specific for the ALFA peptide and additionally with an anti-TUBULIN or anti-GRP78 (an endoplasmic reticulum chaperone) as protein loading controls. First, the ALFA-tagged proteins were detected only in the respective ALFA-tagged strains (Figure 4A–C), whereas the loading control was present in all the worm lysates. Second, the molecular weight of the ALFA-tagged proteins was in agreement with the predicted size for each protein (*Cel-*DLG-1 = 107 kDa; *Ppa-*SULT-1 = 53.3 kDa; *Ppa*-AIN-1 = 70 kDa), highlighting the specificity of the anti-ALFA nanobody. Third, we only observed a cross-reaction of the anti-ALFA nanobody with an endogenous protein of about 55 kDa in *P. pacificus* PS312 lysates following long exposures to the chemiluminescent signal (data not shown). However, the non-specific signal was always much weaker than the one observed for the ALFA-tagged proteins. Thus, the ALFA system is a reliable and straightforward approach to evaluate and quantify the levels of endogenous proteins in nematode model organisms.

### 3.5. Capture of ALFA-Tagged Proteins from Worm Lysates

Affinity purification coupled with mass spectrometry is a commonly used approach to purify protein complexes from cells. We applied the ALFA system to capture, for the first time, an ALFA-tagged protein and its associated proteins in a multicellular organism. We incubated *P. pacificus* PS312 and *Ppa-ain-1(tu1753*[*ain-1*::2xALFA]) worm lysates with the anti-ALFA nanobody immobilized on an agarose-based resin. After washing, the nanobody-bound proteins were competed with an excess of free ALFA peptide. The resulting eluted proteins were analyzed by SDS-PAGE and immunoblotting (Figure 4C). The *Ppa-*AIN-1-2xALFA protein successfully bound to the ALFA resin and was specifically eluted following the incubation with the free ALFA peptide (Figure 4C, upper panel, lane 4). In contrast, TUBULIN did not bind to the resin and therefore was only detected in the worm lysates (Figure 4C, bottom panel, lanes 1 vs. 2 and 3 vs. 4).

To study the efficiency of the ALFA system in the capture of ALFA-tagged proteins from worm lysates, we used immunoblotting and tracked the *Ppa-*AIN-1-2xALFA protein along the affinity purification protocol (Figure S1A,B). Once again, the *Ppa-*AIN-1-2xALFA present in the worm pellet bound to the ALFA resin and was specifically eluted with an excess of free ALFA peptide (Figure S1B, lanes 1 and 5). Nevertheless, we observed that two rounds of incubation with an excess of ALFA peptide was not sufficient to elute all the ALFA-tagged protein bound to the ALFA resin. In fact, enhanced elution of the *Ppa-*AIN-1-2xALFA protein was achieved after an additional incubation of the ALFA resin with a denaturing SDS sample buffer (Figure S1A,B). Thus, improved elution steps can provide higher yields of the protein captured using the ALFA system. Future experiments will indicate whether the efficiency of elution using the ALFA system depends on the ALFA-tagged protein or solely on the ALFA resin.

To examine the specificity and sensitivity of the ALFA system in the isolation of protein complexes in *P. pacificus*, we analyzed the elute fractions from PS312 and *Ppa-*AIN-1::2xALFA by mass spectrometry. We specifically searched for peptides corresponding to the *P. pacificus* homolog of the Argonaute protein, *Ppa-*ALG-1, a known interactor of AIN-1/ GW182 proteins [33]. In comparison to the PS312 sample, *Ppa-*ALG-1 protein was 2.6x fold enriched in the pulldown sample of AIN-1::2xALFA (Figure 4D). As expected, *Ppa-*AIN-1 peptides were only detected in the *Ppa-*AIN-1::2xALFA samples. These results indicated that the ALFA system can also be applied in nematodes to specifically purify a POI and its binding partners.

In conclusion, this study reports the versatile application of the ALFA-tag epitope in the two nematode model organisms *C. elegans* and *P. pacificus*. With their rapid and simple growth and hermaphroditic mode of reproduction, epitope-tagged worms can easily be generated and used for genetic and biochemical characterization. Combined with the advantages of using fluorophore-labelled nanobodies in microscopy [41], the small size and the unique sequence of the tag, our work shows that the ALFA system is suitable for studying the spatial distribution and expression of endogenous proteins in a multicellular organism with a high resolution. In addition, we were able to reproducibly and specifically detect, quantify and capture POIs. We envision that the ALFA tag will be particularly powerful in multiple biochemical and cellular applications. These include genome-scale mapping of protein–protein interactions, protein–DNA/chromatin, and protein–mRNA/small RNA/ncRNA interactions coupled with mass spectrometry and DNA/RNA sequencing approaches, and the sorting of cells carrying surface tagged proteins. The combination of different small tags and respective nanobodies may also open an opportunity to simultaneously visualize multiple proteins in situ. Thus, the ALFA-tag adds another powerful tool to the already existing toolkit of *C. elegans*.

## 4. Limitations of the Current Study

There are two limitations in the current study that can only be addressed through additional applications. First, it remains unknown if C-terminal tags will be applicable in the majority of proteins. In particular, if functional domains in the C-terminus of a POI exist, an ALFA-tag in its proximity might interfere with protein function. At the same time, it is one of the advantages of the ALFA-tag that, in principle, can be added in any position of the protein. Second, for biochemical applications, the pulldown of additional proteins and/or RNAs from a macromolecular complex labeled through ALFA-tagging of one of its protein components, might depend on the expression level of the POI. While the protein used in this study is highly abundant and expressed broadly in most cells of the worm, many POIs are known to have restricted expression patterns. Therefore, future studies will indicate if there are any limits for these types of applications. However, the increasing sensitivity of HPLC/MS-MS analysis for protein identification and the sequencing depth for RNA molecules are likely to help in overcoming these shortcomings.

## Figures and Tables

**Table 1:** Organisms/strains used in this study.

Strain	Source	Modified Gene/ID	Comment
*Caenorhabditis elegans*			
N2 (wild type)	*C. elegans* Genetics Center	--	--
RS4102	Sommer Lab	*unc-10(tu1781*[*unc-10*::2xALFA])	C-terminally tagged *Ce* UNC-10
RS4103		*dlg-1(tu1782*[*dlg-1*::2xALFA])	C-terminally tagged *Ce* DLG-1
*Pristionchus pacificus*			
312 (wild type)	Sommer Lab	--	--
RSC011 (wild type)		--	--
RS4031		*eud-1(tu1729*[*eud-1*::2xALFA])	C-terminally tagged *Ppa* EUD-1
RS4068		*ain-1(tu1753*[*ain-1*::2xALFA])	C-terminally tagged *Ppa* AIN-1
RS4085		*sult-1(tu1760*[*sult-1*::2xALFA])	C-terminally tagged *Ppa* SULT-1

## Supplementary Materials

The following supporting information can be downloaded at: https://www.mdpi.com/article/10.3390/cells11233875/s1, Figure S1: Affinity purification of ALFA-tagged *Ppa-*AIN-1; Table S1: CRISPR/Cas9 sgRNA target sequences and ssDNA donor used in this study; Table S2: Primer sequences used in this study.
