# Supplementary material for: Application of ALFA-Tagging in the Nematode Model Organisms Caenorhabditis elegans and Pristionchus pacificus
Source: Cells. 2022 Dec 1;11(23):3875. doi: 10.3390/cells11233875 (PMC9740511; doi:10.3390/cells11233875)
Supplement: Supplementary file 1 [file cells-11-03875-s001.zip › Supplementary Information/Supplementary Information.pdf]

Supplementary Information

Application of ALFA-tagging in the nematode model organisms *Caenorhabditis elegans* and *Pristionchus pacificus*

Catia Igreja, Tobias Loschko, Alejandra Schäfer, Radhika Sharma, Shiela Pearl Quiobe, Elbin Aloshy, Hanh Witte & Ralf J. Sommer*

Department for Integrative Evolutionary Biology, Max Planck Institute for Biology Tübingen, Max Planck Ring 9, 72076 Tübingen, Germany

*Author for correspondence at: ralf.sommer@tuebingen.mpg.de

Running head: Nematode ALFA-tagging

ORCID numbers:

Catia Igreja: https://orcid.org/0000-0003-3563-1788

Ralf J. Sommer: https://orcid.org/0000-0003-1503-7749

Igreja *et al.* Supplementary Figure 1.

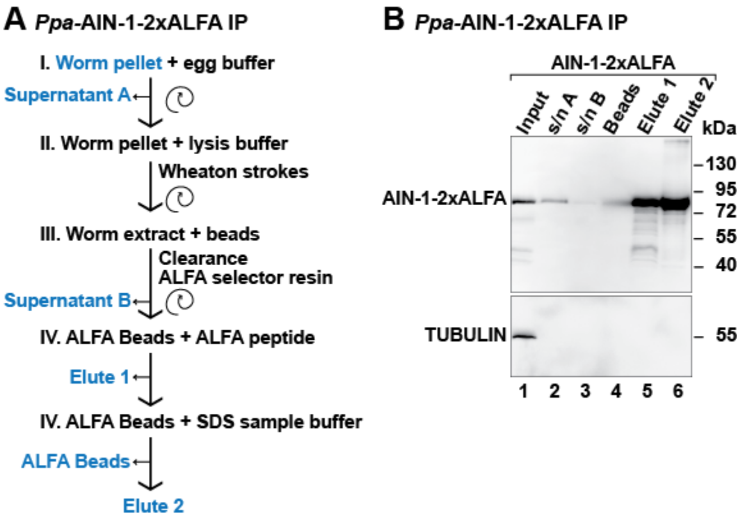

Figure S1. Affinity purification of ALFA-tagged *Ppa*-AIN-1

(A) Simplified schematic representation of the experimental protocol used to capture ALFA-tagged proteins from worm lysates (for details see Materials & Methods section). Curved arrows represent centrifugation steps. Aliquots of samples highlighted in blue were collected throughout the purification steps and analyzed by immunoblotting in B.

(B) Detection of AIN-1-2xALFA by Western blotting. Samples were collected throughout the affinity purification of *Ppa*-AIN-1::2xALFA using ALFA resins. Input: Worm pellet (Input; 10% of worm pellet dissolved in SDS sample buffer), supernatant A (s/n A; 0.1% of worm pellet dissolved in 10 ml of egg buffer), supernatant B (s/n B; 0.1% of worm pellet lysed in 8 ml of lysis buffer), elute 1 (7.5% of ALFA-tagged protein eluted from the ALFA resin following incubation with 400 μM of ALFA peptide); elute 2 (10% of ALFA-tagged protein eluted from the ALFA resin with denaturing SDS sample buffer after collection of elute 1) and beads (0.5% of the ALFA resin at the end of the two elution steps, i.e., ALFA-tagged protein still bound to the beads at the end of the affinity purification protocol) samples were analyzed by SDS-PAGE and Western blot. The membrane was first incubated with anti-TUBULIN primary antibody and corresponding secondary antibody coupled with HRP. After acquisition of the TUBULIN signal, ALFA-tagged protein was detected using the anti-ALFA nanobody.

Table S1. CRISPR/Cas9 sgRNA target sequences and ssDNA donor used in this study

Locus	sgRNA	ssDNA donor
Ce-dlg-1 (C25F6/ G5ECY0)	5'-TGAATCGCAGACGCCAATTT-3'	5'-AGTGTACTCCATCATCAGCCG**TGAATCACAAACACCGATCT**GGGTGCCACGTCAT**TCACGACTCGAAGAAGAACTCCGACGACGACTTACAGAATCCCGTCTCGAGGAGGAGCTCCGTCGTCGTCTCACCGAGTAG**ATGAAATTGGATATTTTTTAGACAAATAATCGTTT-3'
Ce-unc-10 (T10A3/ Q22366)	5'-AAAGATTCCGATGTATCAGT-3'	5'-GCTGGCACTGGTCCAGTTCGA**AAAGATTCAGACGTTTCCGT**CGGAGGTGCTCAGCAG**TCACGACTCGAAGAAGAACTCCGACGACGACTTACAGAATCCCGTCTCGAGGAGGAGCTCCGTCGTCGTCTCACCGAGTAA**CAAATTTCATATGTTTTTGTTTGTTTTTTGTCTTA-3'
Ppa-eud-1 (PPA43535/ A0A4X3PQV1)	5'-TCGAGAAGGAGGAGCTCAA-3'	5'-CGCGCTTCGAAGAGCCATGCTCGAGAAGGAGGAGCTCAA**TTG**GAGTGATCAAGAATACGATCAGCTTGAA**TCTAGACTCGAGGAGGAGCTCAGAAGAAGACTCACTGAGTCCCGTCTCGAGGAGGAGCTCCGTCGTCGTCTCACCGAGTAA**GAATAAATATACCTAACGATTTATCTCGCTTCCATCAGTTC-3'
Ppa-ain-1 (PPA22409/ H3FJF8)	5'-CAACTATCAGTACTCCTTCC-3'	GTCTCAGAGTCGGCCCACGA**CAACTATCAGTATTCGTTCTT**GGGT**TCTAGACTCGAGGAGGAGCTCAGAAGAAGACTCACTGAGTCCCGTCTCGAGGAGGAGCTCCGTCGTCGTCTCACCGAGTAA**GCATCGATGATCTTTGATCTATCGACTCTTCCCTTTCCCCTTC
Ppa-sult-1 (PPA12547/ H3ERY0)	5'-GAAGATCGACGACGCTACAG-3'	5'-TCAATTTCCCTTTGACTCCCTGAAGATCGACGACGCTACAG**AAG**AGCAGGAC**TCTAGACTCGAGGAGGAGCTCAGAAGAAGACTCACTGAGTCCCGTCTCGAGGAGGAGCTCCGTCGTCGTCTCACCGAG**TAATCTTATTTTATCACTTATGTATTCCATTAGAT-3'

Gene names are also identified by the corresponding Wormbase (*Ce*) or El Paco_v3 (*Ppa*) and Uniprot IDs. In the ssDNA donor, the two copies of the Alfa tag are highlighted in bold characters, the STOP codon is depicted in red and the modified PAM motif or gRNA is shown in green.

Table S2. Primer sequences used in this study

Locus	Forward primer	Reverse primer	Sequencing primer
Ce-dlg-1	5’-TTTGTTCACGCGTATGTCTTT-3’	5’-GAACTTGGAAAGGTCATGTAA-3’	Forward primer
Ce-unc-10	5’-TGGAGGGCAAACAATGCATTGC-3’	5’-CAAGTACCCATAATGCATAACG-3’	Forward primer
Ppa-eud-1	5’-CACTTCAACGCATCTGTTAT-3’	5’-AAGCGAGATAAATCGTTAGG-3’	Forward primer
Ppa-ain-1	5’-GATGACGACAGCTAAGATCG-3’	5’-AAATCGGAACGATAATAGGG-3’	Forward primer
Ppa-sult-1	5’-CCCGCTGTGGAGGGTCCGCTGG-3’	5’-ACATTGCTCGTGGAGCAAAAGC-3’	Forward primer
