# Supplementary figures and images for: Application of ALFA-Tagging in the Nematode Model Organisms Caenorhabditis elegans and Pristionchus pacificus
Source: Cells. 2022 Dec 1;11(23):3875. doi: 10.3390/cells11233875 (PMC9740511; doi:10.3390/cells11233875)

Igreja et al. Figure 4 - source data.

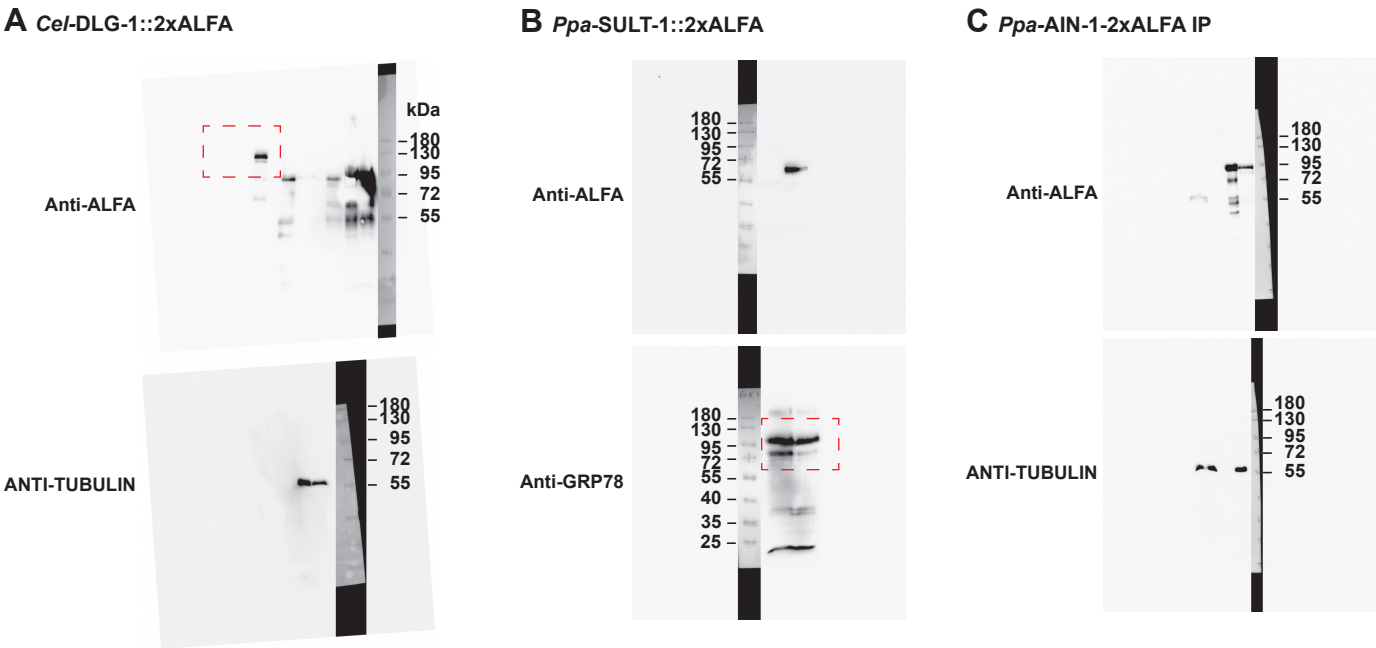

Igreja et al. Supplementary Figure 1 - source data.

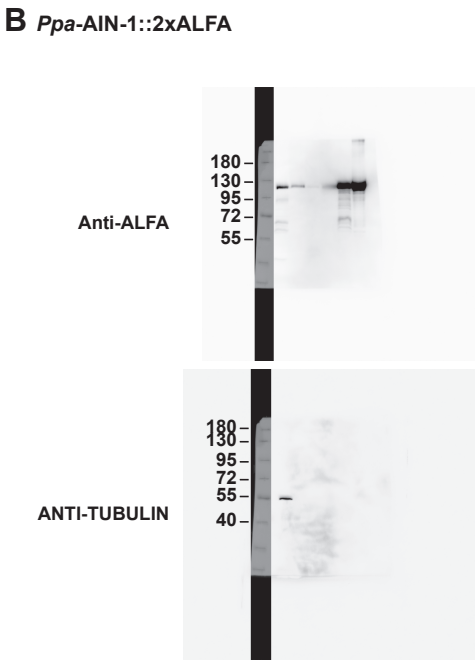

Supplement: Supplementary file 1 [file cells-11-03875-s001.zip › Supplementary Information/source data.pdf]
